# A Synthetic Peptide with the Putative Iron Binding Motif of Amyloid Precursor Protein (APP) Does Not Catalytically Oxidize Iron

**DOI:** 10.1371/journal.pone.0040287

**Published:** 2012-08-14

**Authors:** Kourosh Honarmand Ebrahimi, Peter-Leon Hagedoorn, Wilfred R. Hagen

**Affiliations:** Department of Biotechnology, Delft University of Technology, Delft, The Netherlands; Biological Research Centre of the Hungarian Academy of Sciences, Hungary

## Abstract

The β-amyloid precursor protein (APP), which is a key player in Alzheimer's disease, was recently reported to possess an Fe(II) binding site within its E2 domain which exhibits ferroxidase activity [Duce et al. 2010, Cell 142: 857]. The putative ligands of this site were compared to those in the ferroxidase site of ferritin. The activity was indirectly measured using transferrin, which scavenges the Fe(III) product of the reaction. A 22-residue synthetic peptide, named FD1, with the putative ferroxidase site of APP, and the E2 domain of APP were each reported to exhibit 40% of the ferroxidase activity of APP and of ceruloplasmin. It was also claimed that the ferroxidase activity of APP is inhibited by Zn(II) just as in ferritin. We measured the ferroxidase activity indirectly (i) by the incorporation of the Fe(III) product of the ferroxidase reaction into transferrin and directly (ii) by monitoring consumption of the substrate molecular oxygen. The results with the FD1 peptide were compared to the established ferroxidase activities of human H-chain ferritin and of ceruloplasmin. For FD1 we observed no activity above the background of non-enzymatic Fe(II) oxidation by molecular oxygen. Zn(II) binds to transferrin and diminishes its Fe(III) incorporation capacity and rate but it does not specifically bind to a putative ferroxidase site of FD1. Based on these results, and on comparison of the putative ligands of the ferroxidase site of APP with those of ferritin, we conclude that the previously reported results for ferroxidase activity of FD1 and – by implication – of APP should be re-evaluated.

## Introduction

**Figure 1:**
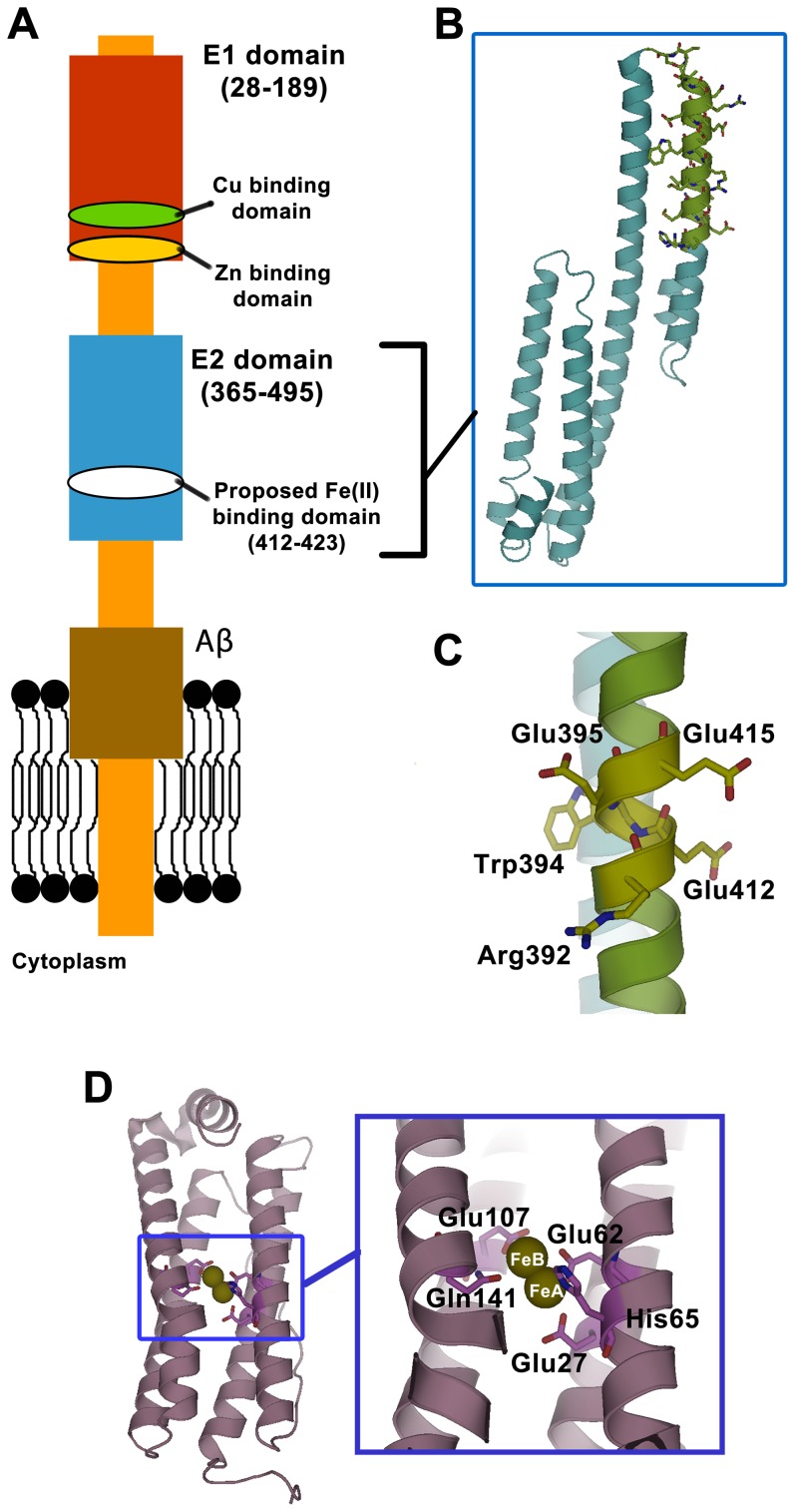
Topology of β amyloid precursor protein (APP) and comparison of its putative ferroxidase site with the ferroxidase site of human H-chain ferritin. (A) A schematic representation of APP and its metal binding domains (APP770 isoform). (B) Structure of the E2 domain of APP751 (PDB 1RW6) [13] and (C) the putative ligands of the ferroxidase site (APP770 numbering). The residues in green show the section of the E2 domain that contains the putative ferroxidase site of APP and the residues that were used to synthesize the FD1 peptide. (D) The diiron catalytic center, the ferroxidase center, of human H-chain ferritin (PDB 1FHA).

Human β-amyloid precursor protein (APP) is generally thought to play a key role in Alzheimer's disease as the source of plaque-forming β-amyloid peptides (Aβ) [1], [2]. APP is a transmembrane protein made up of a large, multidomain extracellular extension, a small, single-pass transmembrane part, and a small intracellular extension (Figure 1A). Alternative exon splicing of the APP gene affords eight different mRNAs that translate into eight APP iso-forms whose length range from 365 to 770 amino acid residues [3], with APP695 as the dominant form in the brain. Sequential processing of APP by the proteolytic enzymes β-secretase and γ-secretase liberates Aβ: a sequence of typically 40 or 42 residues originally located partially in the membrane and for the remainder extracellularly. It is not known whether Aβ in Alzheimer's disease is a causative agent or a resulting product. The physiological role(s) of APP in healthy cells has also not been firmly established.

Metal ions, notably iron, copper, and zinc have been implicated in the normal physiological functioning of APP [4], [5]–[6], in the regulation of APP expression [7], [8], in the processing of APP affording Aβ [8], [9], and in Aβ-plaque related pathogeny [5], [10]. The complete APP has not been crystallized, but the 3D structure of two extracellular domains, E1 and E2, have been determined. Two subdomains of the N-terminal E1 domain, the ‘growth factor like domain’ GFD and the ‘copper binding domain’ CuBD, have separately been crystallized [11], [12], [13]. In none of these four crystal structures any metal has been found. When the CuBD crystal is soaked in 100 mM CuCl_2_ a single site is found with Cu^2+^ square-pyramidal coordination by His147, His151, Tyr168 and two waters [12]. A physiological role, if any, of this copper-binding site has yet to be established. Other putative copper (and zinc) binding sites have been proposed in E1 [5], and physiological studies have implicated copper in APP trafficking [4].

An interaction of iron and the Aβ domain of APP has been reported [14] but a direct interaction with the E2 domain of APP has not been explicitly considered until the recent proposal that APP exhibits an Fe(II) binding site within the E2 domain (Fig. 1B–C). It was proposed that this site has homology with the ferroxidase center in the iron-storing ferritins (Fig. 1D), and it was claimed that the site possesses catalytic iron oxidation activity (ferroxidase activity) [15]. Steady-state kinetics of ferroxidase activity were reported for APP695, for the isolated E2 domain, for a synthetic 22 amino acid peptide named FD1 (duplicating a stretch in the E2 domain with the putative ferroxidase site), and for the human copper protein ceruloplasmin (as a positive control) [15]. Ferroxidase activity of APP was proposed to be functionally relevant in interaction with the iron exporter ferroportin to prevent intracellular iron accumulation and associated oxidative stress. Like in ferritins [16]–[20], the APP ferroxidase activity was reported to be inhibited by binding of zinc to the APP, its E2 domain, or the FD1 peptide [15]. The present study, which elaborates on the proposed ferroxidase activity of the FD1 peptide [15], was incited by five distinct aspects of the previous work: (i) the ferroxidase activity was measured with what appears to be an unvalidated procedure involving transfer of Fe(III) to the iron transport protein transferrin, and reported activities for ceruloplasmin, APP, the E2 domain of APP, and the FD1 peptide were claimed to be orders of magnitude higher than ferritin ferroxidase; (ii) reported Michaelis-Menten plots for ferroxidase activity of each of the four studied proteins had shapes that appeared to be incompatible with the hyperbolic shape of Michelis-Menten kinetics, and V_max_ values that were read as saturation values from the data in figures differed by circa 40% from the values obtained from fitting the Michaelis-Menten equation to the non-hyperbolic data sets; (iii) the k_cat_ value reported in the figures for the FD1 peptide was identical to the values reported for APP and ceruloplasmin, and it was not 40% of the k_cat_ values reported for APP and ceruloplasmin as claimed in the text; (iv) reported ferroxidase activities of the E2 domain for two experiments performed under the same conditions differed by circa 50% while error bars of <1% were claimed(v) the proposed homology between APP and human H-chain ferritin appeared to be based on a mistake of assignment of iron coordinating amino acids [15]. We have therefore re-evaluated the transferrin-based ferroxidase assay by comparison with direct measurements of O_2_ consumption, and we have compared ferroxidase activity of the putative ferroxidase peptide FD1 from APP with those of ceruloplasmin and human H-chain ferritin.

## Results

### Significance of non-enzymatic oxidation of Fe(II) in the presence of transferrin

**Figure 2:**
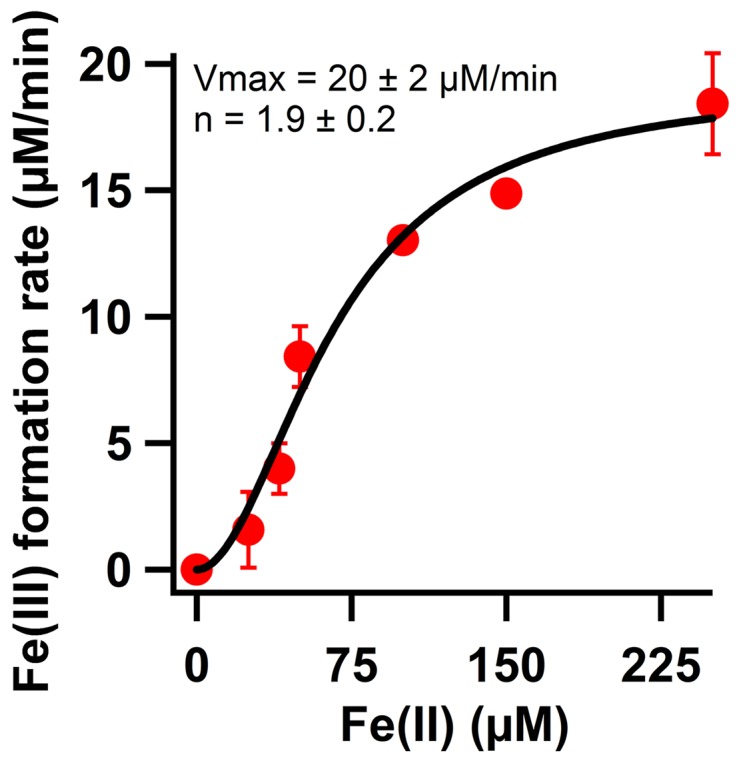
Effect of concentration of Fe(II) on non-enzymatic oxidation of Fe(II) and incorporation of the Fe(III) product into transferrin. Progress curves were recorded at 460 nm for formation of diferric-transferrin and the initial rates were plotted versus concentration of Fe(II). The error bars represent standard deviation of three independent measurements. Concentration of apo-transferrin was 100 µM. Buffer was 100 mM Hepes, 100 mM NaCl, pH 7.2. Measurements were performed at 37°C.

Osaki [21], [22] used the Fe(III) binding protein transferrin to study the ferroxidase activity, i.e. enzymatic oxidation of Fe(II) by molecular oxygen, of ceruloplasmin. In the absence of ceruloplasmin and for an Fe(II) concentration of 100 µM, the overall initial rate of non-enzymatic oxidation of Fe(II) by molecular oxygen and subsequent incorporation of the Fe(III) product into transferrin at pH 7.3 was significant, namely, 24 µM Fe(III) formed per minute [22]. More recently, Duce et al used transferrin in attempts to test the ferroxidase activity of human β-amyloid precursor protein (APP), of the E2 domain of APP, and of a 22-residue sub-domain of E2 in the form of the synthetic peptide, FD1 [15]. In that study the initial rate of non-enzymatic oxidation of Fe(II) in the presence of transferrin was reported to be zero for an Fe(II) concentration of 0–200 µM and at pH 7.2. This observation contradicts the original results reported by Osaki. Therefore, we checked the significance of non-enzymatic oxidation of Fe(II) and incorporation of the Fe(III) product into transferrin at pH 7.0 in the absence of any other proteins and at various concentrations of Fe(II) (Fig. 2). Kinetics with apparent positive cooperativity was observed and the Hill equation was used to fit the data with a Hill constant of 1.9 and a V_max_ of 21 µM/min. The observed V_max_ is comparable to all previous reported values by others [21]–[23], and is within experimental error comparable to the values reported by Duce et al [15] for the putative ferroxidase activity of the APP protein, the E2 domain, and the FD1 peptide. The rate of non-enzymatic oxidation of Fe(II) and formation of diferric-transferrin dramatically increased with increasing the pH from 6.2 to 8.2. The initial rate of Fe(III) formation (µM per minute) was 0.08±0.01 at pH 6.2, 2.6±0.4 at pH 7.2, and 80.2±8.5 at pH 8.2 for an initial Fe(II) concentration of 40 µM. Thus, the initial rate of non-enzymatic oxidation of Fe(II) in the presence of transferrin depends on the concentration of Fe(II) and on the pH.

### The use of transferrin to study the ferroxidase activity of H-chain ferritin and ceruloplasmin

**Figure 3:**
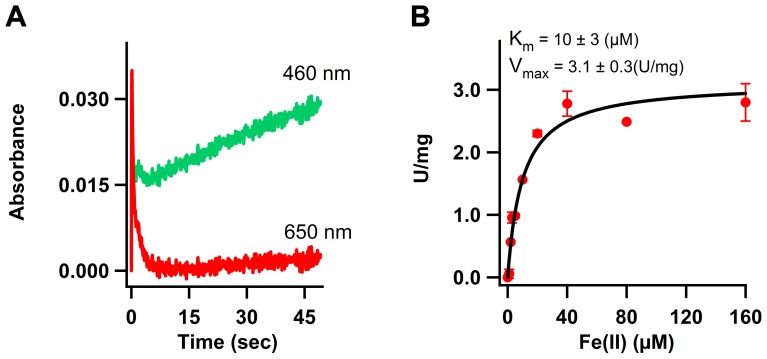
Use of transferrin to measure the ferroxidase activity of ferritin and ceruloplasmin. (A) Effect of transferrin on Fe(II) oxidation by human H-chain ferritin (HuHF) measured at 650 nm for formation and decay of the blue intermediate, and at 460 nm for formation of diferric transferrin complex. Concentration of apo-HuHF was 1.7 µM (24-mer) and that of transferrin was 70 µM. Two Fe(II) per ferroxidase center, i.e. 48 Fe(II) per 24-mer which is equal to a final concentration of 81.6 µM Fe(II), were added and measurements were done at 34°C in 400 mM Mops buffer, 100 mM NaCl, pH 7.0. (B) Ferroxidase activity of ceruloplasmin was measured using the transferrin assay at 460 nm. Progress curves were recorded and initial rate of Fe(III) formation was plotted versus concentration of Fe(II). Concentration of ceruloplasmin was 0.1 µM. Measurements were performed at 37°C in triplicate. Buffer was 100 mM Mops, 100 mM NaCl, pH 7.1.

Before using the transferrin assay for measurement of the ferroxidase activity of the FD1 peptide with the putative ferroxidase site of APP, we tested this assay by measuring the established ferroxidase activities of human H-chain ferritin (HuHF) and ceruloplasmin. Bakker and Boyer measured the iron oxidation activity of horse spleen ferritin (HoSF) at pH 6.2 in the presence of human transferrin [23]. They reported the activity as formation of diferric-transferrin (i.e. sequestering of Fe(III) by transferrin from ferritin) under the – then unproven – assumption that the ferroxidase activity of ferritin was the rate-limiting step in the overall assay, and under the assumption that all the 24 subunits of HoSF were catalytically active. Subsequent studies have shown that HoSF consists of two distinct subunits, 80% L ‘light’ and 20% H ‘heavy’ subunits [24]; and the catalytic center is only present in the H subunit [25], [26]. Measurements of kinetics of iron oxidation by HuHF have shown that oxidation of Fe(II) occurs within seconds [27]. In this process two Fe(II) bind simultaneously to the catalytic site of ferritin, which is commonly known as the ferroxidase site (FC), and they are oxidized by molecular oxygen at a high rate. This reaction has typically been measured by following the UV-visible absorbance spectrum of ferritin-bound Fe(III) between 300 nm and 500 nm [28]. Using this procedure a k_cat_ = 3.3 sec^−1^ has been reported for HuHF [26] which is comparable to the values reported by Duce et al for ceruloplasmin and not orders of magnitude lower as claimed by them [15]. We determined whether the oxidation rate of Fe(II) by the FC of HuHF in one turnover, i.e. oxidation of 2 Fe(II) per FC, is comparable to the rate of diferric-transferrin complex formation. Absorbance at 650 nm was recorded for the formation and decay of a blue intermediate, the proposed μ-peroxodiferric complex, which is an indicator of the oxidation of Fe(II) in the ferroxidase site of HuHF [29], [30], and at 460 nm for the formation of diferric-transferrin complex. An amount of 2 Fe(II) per FC was added to apo-HuHF in the presence of transferrin and, using a stopped-flow spectrometer, the spectra were recorded (Fig. 3A). Within five seconds the blue intermediate with maximum absorbance at 650 nm forms and decays. This rate of formation and decay for the blue intermediate is comparable to the previously reported values for HuHF [29]. The rise in absorbance at 460 nm in the first five seconds is due to the broad absorbance spectrum of ferritin-bound Fe(III) from the UV region to circa 500 nm [28]. Subsequently, the absorbance at 460 nm slowly increases because transferrin scavenges ferritin-bound Fe(III). Thus the rate limiting step is the transfer of the Fe(III) from ferritin to transferrin, and – contrary to the proposal of Bakker and Boyer [23] – the rate of diferric-transferrin complex formation under these conditions is not equal to the rate of the ferroxidase activity. Therefore, the transferrin assay is not a suitable method to quantitatively measure ferroxidase kinetics of the iron-storage protein ferritin.

Then, we measured the ferroxidase activity of ceruloplasmin as a function of Fe(II) concentrations using the transferrin assay (Fig. 3B). The specific activity of ceruloplasmin was calculated from the initial rate of diferric-transferrin formation which was obtained from the progress curves at 460 nm using an extinction coefficient of 4.56 mM^−1^ cm^−1^ for the diferric-transferrin complex. The activity was measured for Fe(II) concentrations between 0 and 160 µM. Addition of ceruloplasmin significantly increased the rate, and at an Fe(II) concentration of 40 µM a maximum rate of 240 µM Fe(III) formed per minute per µM of ceruloplasmin was observed (Fig. 3B). The data were fitted to the Michaelis-Menten equation affording K_m_ = 10±3 µM and V_max_ = 3.1±0.3 U/mg (µmol of Fe(II) oxidized per minute per mg of ceruloplasmin). It can be concluded that unlike HuHF, ceruloplasmin does not store iron and the resulting Fe(III) product of the ferroxidase reaction is rapidly scavenged by transferrin and therefore the transferrin assay can be used to measure the activity of ceruloplasmin.

### The FD1 peptide does not have ferroxidase activity in the transferrin-coupled assay

**Figure 4:**
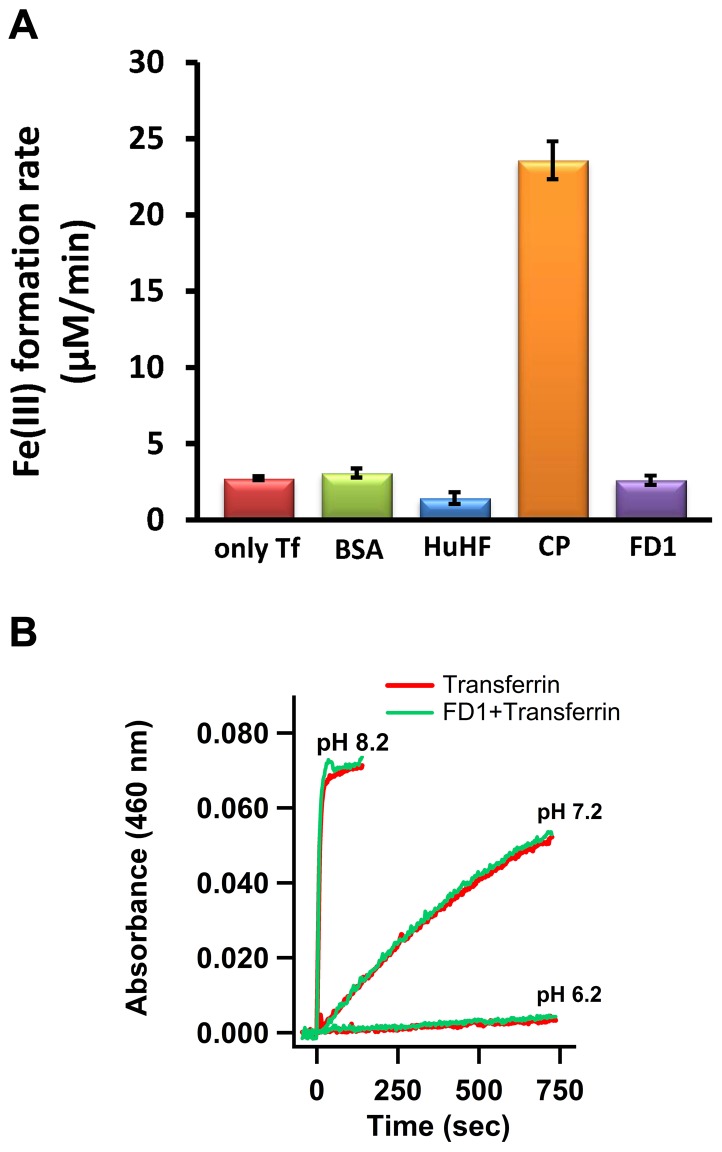
The FD1 peptide does not catalytically oxidize iron as measured by the transferrin assay. (**A**) Non-enzymatic oxidation of Fe(II) and incorporation of Fe(III) into transferrin was compared with that of BSA, HuHF, ceruloplasmin (CP) and FD1. 52 µM of apo-transferrin was mixed with 2 µM BSA or 0.18 µM HuHF (24-mer) or 2 µM FD1 peptide, or 0.1 µM ceruloplasmin, and then an aliquot of 5 µl anaerobic solution of ferrous sulphate was added. Measurements were done in 100 mM Hepes, 100 mM NaCl, pH 7.2. Final concentration of Fe(II) was 40 µM. (B) Effect of pH on non-enzymatic oxidation of Fe(II) in the presence of transferrin was checked at three different pH values in the presence or absence of FD1 peptide (2 µM): pH 6.2, 100 mM Mes, 100 mM NaCl; pH 7.2, 100 mM Hepes, 100 mM NaCl, and pH 8.2, 100 mM Tris, 100 mM NaCl. Concentration of apo-transferrin was 52 µM. Final concentration of Fe(II) was 40 µM. Measurements were performed at 37°C in triplicate with two different batches of transferrin.

The reported ferroxidase activity of APP was tested using the 22-residue synthetic peptide FD1, which carries the putative ferroxidase active site of APP. Duce et al have reported that this peptide possesses 40% of the activity of APP or ceruloplasmin [15]. The initial rate of diferric-transferrin complex formation due to oxidation of Fe(II) by FD1 was measured and was compared (i) to that of the non-enzymatic oxidation in the presence of transferrin, (ii) to that of BSA which is known not to oxidize Fe(II), (iii) to that of HuHF which should decrease the rate because it stores the Fe(III), and finally (iv) to that of ceruloplasmin (Fig. 4A). At an Fe(II) concentration of circa 40 µM the rate of the non-enzymatic reaction is circa 3 µM Fe(III) formed per minute (Fig. 4A and see Fig. 2). Ceruloplasmin shows a rate that is 8-fold above that of the non-enzymatic reaction. This rate of 24 µM Fe(III) per minute (i.e. a specific activity of 3.1 U/mg) is comparable to the originally reported value for this protein [22]. With the FD1 peptide, however, the rate of diferric-transferrin formation is within experimental error equal to that of non-enzymatic oxidation of Fe(II) by molecular oxygen either in the presence or in the absence of BSA. To test if the pH can have an effect on the rate of Fe(II) oxidation by FD1, the ferroxidase activity of FD1 was examined at three different pH values, 6.2, 7.2, and 8.2 (Fig. 4B). The results were within experimental error identical to those of the non-enzymatic oxidation of Fe(II). The presence of FD1 did not change the rate of iron oxidation and diferric-transferrin complex formation at any of the tested pH values.

### The FD1 peptide does not have oxygen-consumption activity in the presence of Fe(II)

**Figure 5:**
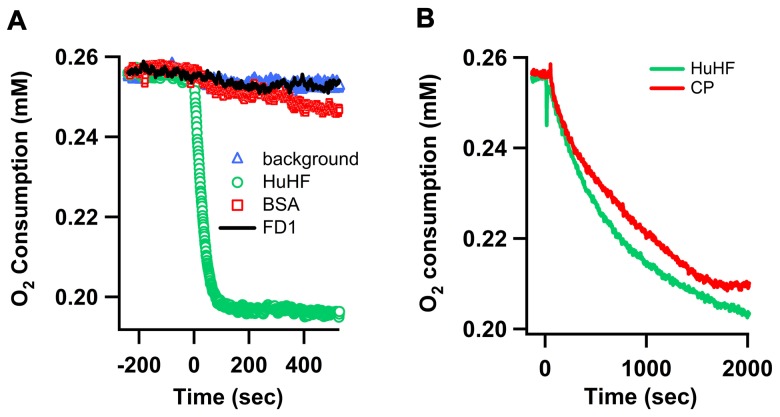
Oxygen consumption as a monitor of Fe(II) oxidation.

A second procedure that has been used to follow the ferroxidase activity of proteins such as ceruloplasmin and ferritin, and one that provides information about the stoichiometry of the reaction, is to amperometrically measure the consumption of the substrate molecular oxygen [31], 32,33. Therefore oxygen consumption by FD1 was measured and was compared with that of ceruloplasmin and HuHF as positive controls. BSA does not consume oxygen and was used as a negative control. It has been shown that when two Fe(II) per subunit are added to HuHF, one molecular oxygen is consumed at a high rate for the oxidation of two Fe(II) in the ferroxidase center [26], [34]. When the amount of Fe(II) added per subunit of ferritin is increased, the Fe(II)/O_2_ ratio increases, and at Fe(II) per subunit ratios greater than 15 circa 4 Fe(II) are oxidized per molecular oxygen [35]. When we added 10 Fe(II) per subunit to HuHF (Fig. 5A) a stoichiometry of 2.4 Fe(II) oxidized per one O_2_ consumed was found, which is in agreement with previous studies [26], [36]. At Fe(II) concentration of 200 µM the rate of oxygen consumption of ceruloplasmin was comparable to that of HuHF (Fig. 5B). As previously reported [22] a stoichiometry of one O_2_ consumed per 4 Fe(II) oxidized was obtained for ceruloplasmin, which suggests that molecular oxygen is reduced to water by ceruloplasmin. Comparison of oxygen consumption of FD1 with that in the non-enzymatic reaction (no peptide or protein present) and that of HuHF, ceruloplasmin and BSA (Fig. 5) provides further independent evidence that FD1 does not have ferroxidase activity. The rate of oxygen consumption in the presence of FD1 is not different from that in the presence of BSA and is equal to non-enzymatic oxidation of Fe(II). From the combined results of iron oxidation and oxygen consumption measurements we conclude that FD1 does not have ferroxidase activity.

### Fe(II) and Zn(II) binding to transferrin and to the FD1 peptide

**Figure 6:**
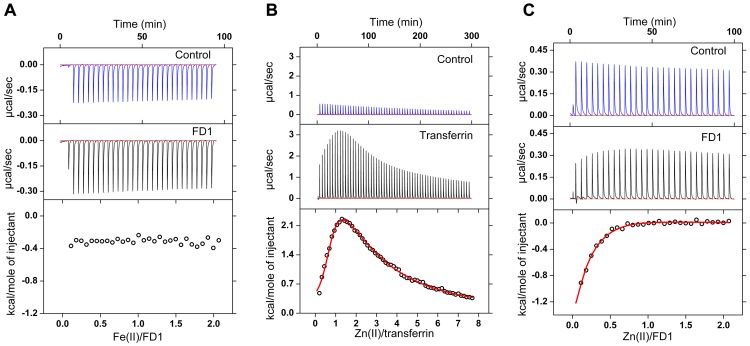
Fe(II) and Zn(II) binding to transferrin and to FD1 measured by isothermal titration calorimetry. (A) Anaerobic Fe(II) titration of the FD1 peptide. Concentration of FD1 in the cell was 70 µM and that of Fe(II) in the syringe was 2.23 mM. The Fe(II) solution was prepared in 100 mM Mops, 100 mM NaCl pH 5.8, and FD1 was in 100 mM Mops, 100 mM NaCl, pH 7.0. The data for the integrated heat of binding were corrected for the heat of dilution due to titration of Fe(II) to buffer (control). Measurements were performed at 25°C. (B) Zn(II) binding to transferrin and (C) to the FD1 peptide. For experiments with transferrin concentration of protein in the cell was 126 µM and the concentration of Zn(II) in the syringe was 12 mM. For experiments with the FD1 peptide, concentration of peptide in the cell was 70 µM and that of Zn(II) in the syringe was 3.35 mM. Transferrin, FD1 peptide, and Zn(II) were prepared in 100 mM Mops, 100 mM NaCl, pH 7.0. Measurements were performed at 25°C. The data for the integrated heat of binding were corrected for the heat of dilution due to titration of Zn(II) to buffer (control).

Because FD1 does not show any iron oxidation activity, we checked if it binds Fe(II) at all. We measured anaerobic binding of Fe(II) to the FD1 peptide using isothermal titration calorimetry (ITC) (Fig. 6A). No Fe(II) binding to the FD1 peptide was observed within the detection limit of ITC.

Then, using ITC binding of Zn(II) to transferrin and to the FD1 peptide was measured (Fig. 6B–C). Statistical analysis of the integrated heat of binding data was performed to obtain the thermodynamic parameters. For transferrin a model of two independent binding sites was required to obtain a fit to the data of integrated heat (Fig. 6B). A binding with K_a1_ = (6.0±1.8)·10^4^ (M^−1^), (Table 1) and with a stoichiometry of circa one Zn(II) per transferrin was found. A second site with a stoichiometry of circa one and with a lower affinity K_a2_ = (1.3±0.6)·10^3^ (M^−1^) (Table 1) was also observed. Zn(II) has been shown to bind to human serum transferrin as well [37]. Previous measurements of Zn(II) binding to transferrin using difference UV monitoring in the presence of competing ligands have suggested two strong binding sites for Zn(II) [38]. Unlike the previous study we measured Zn(II) binding to transferrin directly in the absence of any competing ligands. For analysis of Zn(II) binding to FD1 peptide (Fig. 6C) a model of a single binding site was sufficient. A binding with K_a_ = (4.1±1.0)·10^4^ M^−1^ and stoichiometry of circa 0.5 Zn(II) per FD1 peptide was obtained. This stoichiometry suggests that two FD1 molecules create one coordinating site for a single Zn(II) (Table 1). The observation that Zn(II) apparently does not bind to the putative ferroxidase site of a single peptide is consistent with the results of a recent X-ray crystal structure determination of monomeric E2 domain of APP in the presence of Zn(II) or Cd(II) in which no metal binding to the ligands of the putative ferroxidase site has been observed [39].

**Table 1:** Thermodynamic parameters for Zn(II) binding to transferrin and FD1 peptide.

	Transferrin	FD1
N_1_	0.8±0.06	0.5±0.2
K_a1_ (M^−1^)	(6.0±1.8)·10^4^	(5.1±1.9)·10^4^
ΔH_1_ (kJ/mol)	−1.2±0.2	−2.5±0.6
ΔG_1_ (kJ/mol)	−27.7±0.5	−26.6±1.0
N_2_	1±0.5	----
K_a2_ (M^−1^)	(1.3±0.6)·10^3^	----
ΔH_2_ (kJ/mol)	45±16	----
ΔG_2_ (kJ/mol)	−17.4±1.2	----

Binding of Zn(II) to transferrin and FD1 peptide was measured by isothermal titration calorimetry. N is the stoichiometry of Zn(II) binding per protein. Measurements were performed at 25°C. A two independent binding site model or a single binding site model was used to fit the data points for integrated heat of binding. Standard deviations were obtained from triplicate experiments.

### Zn(II) bound to transferrin inhibits binding of the Fe(III) product of Fe(II) oxidation

**Figure 7:**
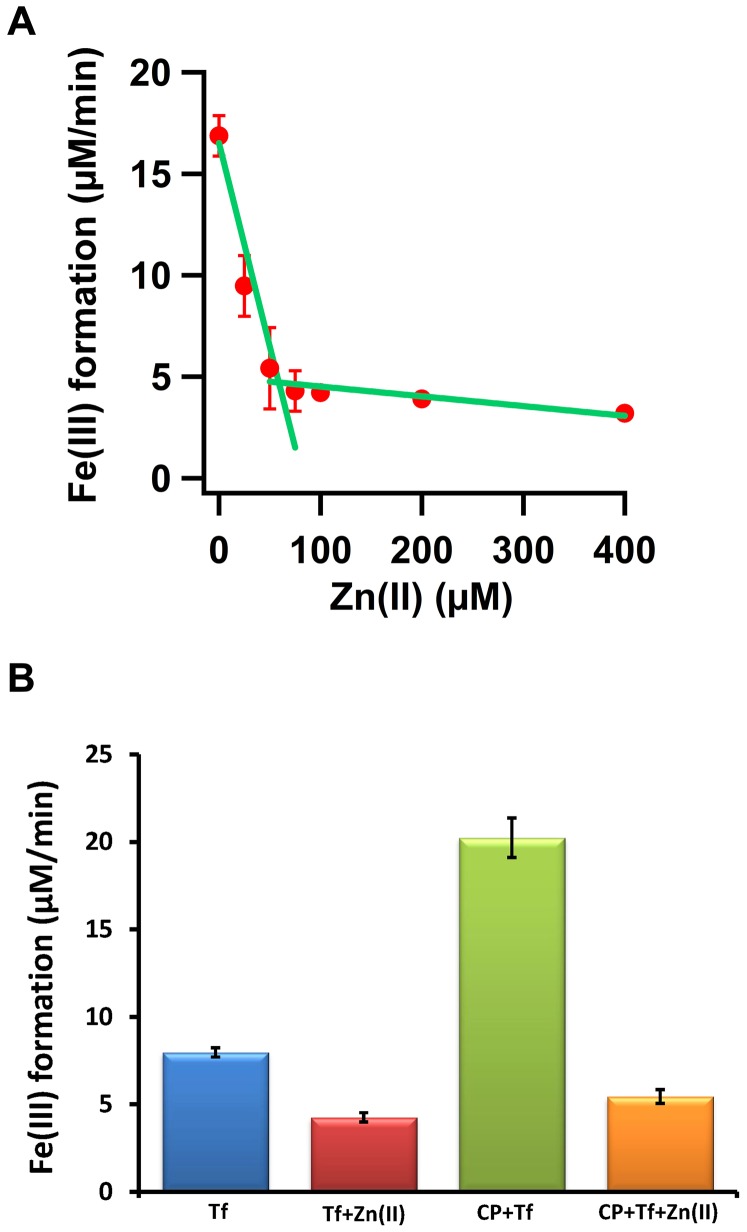
Inhibitory effect of Zn(II) on diferric-transferrin complex formation. (A) Initial rates of diferric-transferrin formation at 460 nm are plotted versus concentration of Zn(II). Concentration of apo-transferrin was 80 µM and that of Fe(II) was 200 µM. (B) Comparison of the inhibitory effect of Zn(II) on the diferric-transferrin complex formation for the non-enzymatic oxidation of Fe(II) by molecular oxygen in the presence of transferrin (Tf 70 µM) and for the ferroxidase activity of ceruloplasmin (CP 0.08 µM) measured with the transferrin assay. Final concentration of Fe(II) was 60 µM and that of Zn(II) was 70 µM. Measurements were done in 100 mM Hepes buffer, 100 mM NaCl, pH 7.2 in triplicate at 37°C.

Because FD1 does not bind Fe(II) and does not catalytically oxidize Fe(II), we surmise that the previously observed inhibitory effect of Zn(II) on the Fe(II) oxidation rate in the presence of transferrin [15] could be due to binding of Zn(II) to transferrin and thus to a decrease in the rate of diferric-transferrin formation. Therefore, we measured the effect of Zn(II) concentration on the rate of non-enzymatic oxidation of Fe(II) (200 µM) and incorporation of the Fe(III) product into transferrin (Fig. 7A). The overall rate decreased considerably in the presence of Zn(II) presumably because Zn(II) binds to the Fe(III) binding sites of transferrin. The addition of one Zn(II) per transferrin was enough to decrease the rate by approximately 80%. The reason for observation of this residual rate in the presence of Zn(II) is possibly the fact that Fe(III) binds transferrin orders of magnitude stronger than Zn(II). Thus Fe(III) can displace Zn(II) from its binding sites. If Zn(II) inhibition of putative ferroxidase activity of APP would be specific, i.e. because of binding of Zn(II) to the APP and not to transferrin, then Zn(II) should not inhibit ferroxidase activity of ceruloplasmin as indeed claimed by Duce et al. [15]. However, we find with the transferrin assay (Fig. 7B) that in the presence of Zn(II) the ferroxidase activity of ceruloplasmin is diminished and is comparable to the residual rate of non-enzymatic oxidation of Fe(II) in the presence of at least one Zn(II) per transferrin. This decrease in rate is apparently due to binding of Zn(II) to the transferrin and inhibition of Fe(III)-transferrin complex formation.

## Discussion

In human H-chain ferritin the ferroxidase reaction occurs in the ferroxidase center made up of five residues [40] that can bind two Fe(II) simultaneously through eight coordination bonds to afford their oxidation by molecular oxygen (Fig. 1). On the contrary, the crystal structure of the E2 domain of APP [13] shows (Fig. 1) that the RExxE putative ferroxidase site of APP [15] consists of only two putative metal-binding residues, namely E412 and E415 in APP770. The side chain of these two residues point to the surface of the protein, i.e. there do not appear to be additional residues with which to form a metal binding site. In the previous work of Duce et al. the two residues E412 and E415, have been arbitrarily aligned with two metal-coordinating residues of the ferroxidase centre of human H-chain ferritin, namely E62 and H65 [15]. However, the two glutamates in the RExxE motif in ferritin are *not* ligands to the metals in the ferroxidase center. In other words, there is no significant overall similarity between the structure of the ferroxidase centre of ferritin and the RExxE putative ferroxidase activity carrying motif of APP.

We argue that the study reported by Duce et al. [15] on ferroxidase activity of purified APP695, its E2 domain, the 22-residue synthetic peptide FD1, and tissues containing APP, and the inhibition of these activities by Zn(II) has several important deficiencies: (i) The authors find that non-enzymatic oxidation of up to 200 µM Fe(II) in the presence of transferrin is negligible at pH 7.2 [15]. This is clearly in disagreement with several previous reports [21]–[23] and with our current results. The rate of non-enzymatic oxidation of Fe(II) by molecular oxygen and incorporation of the resulting Fe(III) product into transferrin is significant at pH 7.2. This rate is not dependent on the presence of the FD1 peptide, indicating that FD1 does not have any ferroxidase activity. Increasing the pH from 6.2 to 8.2 drastically increases the rate of non-enzymatic oxidation of Fe(II) and formation of diferric-transferrin. The effect of pH is crucial since results based on small differences or changes in the pH could easily be mistaken for enzymatic activity. We find that the overall non-enzymatic rate of oxidation of Fe(II) and incorporation of the Fe(III) product into transferrin at pH 7.0 is within experimental uncertainty close to the previously claimed specific activity values for APP, its E2 domain, and FD1 [15]. (ii) In the previous study the fact that Zn(II) can bind to transferrin and inhibit the formation of the diferric-transferrin complex has not been considered [15]. It has been suggested that Zn(II) binds to the putative ferroxidase site of APP but not to ceruloplasmin and that it inhibits ferroxidase activity of APP only. However, in agreement with other previous studies [38], [41] we have shown that Zn(II) binds to transferrin. Binding of Zn(II) to transferrin inhibits formation of the diferric-transferrin complex and therefore decreases the ferroxidase activity of ceruloplasmin in the transferrin assay. (iii) Duce et al. have not measured oxygen consumption by APP as an independent method for ferroxidase activity assay and for determination of the stoichiometry of the ferroxidase reaction of APP, however, they have proposed a model in which APP can catalyse the oxidation of four iron by one molecular oxygen [15]. The oxygen consumption measurements reported here show that FD1 does not consume oxygen in the presence of iron. (iv) Duce et al. have not measured Fe(II) binding to APP, its E2 domain or FD1 peptide. Using isothermal titration calorimetry we observed that the putative catalytic site of the FD1 peptide does not bind Fe(II).

We therefore conclude that the FD1 peptide with the RExxE ferroxidase consensus motif of APP does not carry ferroxidase activity. In view of these results and of the observation that Zn(II) binding to transferrin directly interferes with the measurement of ferroxidase activity using this protein, we suggest that the results of Duce et al [15] for the ferroxidase activity of the FD1 peptide and – by implication – 0f the E2 domain and the full-lenght APP should be re-evaluated.

## Materials and Methods

### Chemicals

All chemicals were reagent grade and were purchased from Sigma Aldrich. Human ceruloplasmin (40 U/mg) was also obtained from Sigma Aldrich.

### Expression and purification of human H chain ferritin

The *Escherichia coli* Top10 strain (Invitrogen) was transformed with the plasmid containing the coding sequence for expression of human H chain ferritin (HuHF) pET12a. After two hours of growth to an OD_600 nm_ of 0.6, production of HuHF was induced by the addition of 0.1 mM IPTG. After eight hours cells were collected and were disrupted using a cell disruptor system (Constant systems). The supernatant was collected using centrifugation and was subjected to a heat step at 85°C for 10 min. Denatured proteins were separated using a centrifugation step. HuHF was made apo as described previously [32] and the final preparation contained less than one iron per 24-meric protein as determined with the ferene assay. The concentration of protein was measured using the bicinchoninic acid (BCA) assay using bovine serum albumin as standard.

### Preparation of apo-transferrin

Apo-bovine transferrin (>98% pure) was purchased from Sigma Aldrich. The lyophilized powder was dissolved in 10 mM Mops, 100 mM NaCl, pH 7.0 and was dialyzed for at least 3 days against the same buffer using dialysis tube (Spectrumlabs) with a cut-off of 10 kDa. Subsequently consecutive dilution and concentration steps using an ultrafiltration membrane with a cut-off of 30 kDa (Millipore) were used to clean transferrin from possible metal complexing molecules which may have remained in the transferrin lyophilized powder from the manufacturing process and to concentrate the protein. The concentration of transferrin was measured using the bicinchoninic acid (BCA) assay and the purified apo-transferrin was directly used in experiments.

### Preparation of the synthetic peptide (FD1)

The peptide FD1 (90% pure, HPLC) was purchased from GenScript. 4 mg of peptide was dissolved in 1 ml of 10 mM Hepes, 100 mM NaCl, pH 7.2, and dilutions were used directly in experiments. The sequence HRERMSQVMREWEEAERQAKNL was confirmed by low energy collision induced dissociation tandem mass spectrometry using a hybrid quadrupole time-of-flight mass spectrometer (Waters QTof Premier).

### Preparation of ferrous sulphate solution

The Fe(II) solution was prepared as explained previously [17], [32].The pH of Milli.Q water was set to 2.5 using HCl. The solution was made anaerobic by flushing with high purity argon gas (99.999%), and then it was added to ferrous sulphate salt in an anaerobic glove box (Coy Laboratory Products).

### Steady state kinetics of Fe(II) oxidation in the presence of transferrin

Kinetics of Fe(II) oxidation and Fe(III) uptake by transferrin were measured at 460 nm using a molar extinction coefficient for diferric transferrin of ε_460 nm_ = 4.56 mM^−1^ cm^−1^ [23]. Measurements were carried out on a fiber-optics spectrophotometer (Avantes) using 1 ml glass cuvettes with a path length of 1 cm. Measurements were done at 37°C in 100 mM Hepes, 100 mM NaCl, pH 7.2 unless otherwise stated. Progress curves were recorded for circa 5 minutes and initial rates were obtained from the slope of a line that was fitted to the data points for the first 50–100 seconds. Each experiment was repeated at least three times with two different batches of transferrin. For kinetic measurements to each cuvette the following additions were made in order (total volume of 1 ml): Hepes buffer, aliquot of transferrin between 50 to 100 µl to reach the desired final concentration, 3.5 µl of 1.4 mM FD1 peptide (as control experiment no peptide was added), and aliquots of FeSO_4_ solution, between 0.5 µl and 20 µl. To determine the inhibitory effect of zinc, aliquots of 0.5 µl to 20 µl of ZnSO_4_ solution were added 5 minutes before addition of FeSO_4_.

### Oxygen consumption measurements

To measure consumption of oxygen a Clark electrode was used as described before [32]. The final reaction volume in the cell was 2 ml. To the cell the following was added in order: buffer, protein or peptide. Subsequently aliquots of 5 µl anaerobic solution of ferrous sulfate were added using a gastight syringe. The buffer was 100 mM Hepes, 100 mM NaCl, pH 7.2. Measurements were performed at 22°C.

### Fast kinetics of Fe(II) oxidation

Fe(II) oxidation by human H chain ferritin was followed using a Scientific PQ/SF-53 preparative quench/stopped-flow instrument with an EG&G Princeton Applied Research 1,024-element photodiode-array detector. The instrument was equipped with a nitrogen gas flow on top of the sample cells to prevent oxidation of the Fe(II) solution before mixing with protein. The instrument was set to zero with rapid mixing of protein with anaerobic water, pH 2.4, before each measurement. The buffer was 400 mM Mops, 100 mM NaCl, pH 7.0.

### Isothermal titration calorimetry

Zn(II) binding to transferrin was measured using isothermal titration calorimetry with a VP-ITC microcalorimeter (GE healthcare). A control experiment in the absence of protein or peptide was performed to measure the heat generated due to Fe(II) or Zn(II) dilution in the buffer. The conditions for the control experiments were identical to the conditions for titration of Fe(II) or Zn(II) to transferrin or to FD1 peptide. For anaerobic Fe(II) binding to FD1, the ITC machine was placed in a polyethylene bag (Atmosbag, Sigma Aldrich). The bag was made anaerobic by high purity argon gas (99.999%). All solutions were made anaerobic using the same argon gas. The Fe(II) solution was prepared in 200 mM Mops, 100 mM NaCl pH 5.8 and the protein was in the same buffer with pH 7.0. Measurements were performed at 25°C in duplicate. The parameter settings of the ITC machine were number of injections 30 or 60, volume of each injection 3 µl, stirring rate 307 rpm, and time spacing 200 sec. The volume of the first injection was 2 µl and the resulting data point was excluded from the fit of integrated heat of binding. The results of ITC were analyzed using Origin 7.0 software under a two independent binding sites model or a single binding site model.
